# Mechanistic investigation of Shuanghuanglian against infectious bronchitis in chickens: a network pharmacology and molecular dynamics study

**DOI:** 10.3389/fvets.2025.1557850

**Published:** 2025-03-12

**Authors:** Fuming You, Hanzhao Zhang, Linghao Meng, Chuanhong Li, Yuxia Yang, Yongqiang Wang, Rigetu Zhao, Luomeng Chao

**Affiliations:** ^1^College of Animal Science and Technology, Inner Mongolia MINZU University, Tongliao, China; ^2^College of Computer Science and Technology, Inner Mongolia MINZU University, Tongliao, China; ^3^Zhalantun Vocational College, Zhalantun, China; ^4^Chifeng Academy of Agricultural and Animal Husbandry Sciences, Chifeng, China; ^5^Inner Mongolia Rambo Testing Technology Limited Company, Tongliao, China; ^6^Inner Mongolia Engineering Technology Research Center for Prevention and Control of Beef Cattle Diseases, Tongliao, China

**Keywords:** infectious bronchitis, network pharmacology, Shuanghuanglian, machine learning, molecular docking, molecular dynamics simulation

## Abstract

**Introduction:**

Infectious bronchitis (IB) poses a major challenge to global poultry production, causing substantial economic burdens and underscoring the necessity for novel therapeutic interventions given the limitations of current vaccines and conventional antiviral agents. The purpose of this study is to comprehensively explore the active components in Shuanghuanglian and their interaction with the key pathological targets of IBV (Infectious bronchitis virus) infection. By using advanced computational methods, this study aims not only to identify the therapeutic potential of active ingredients, but also to reveal their mechanism of action against IBV.

**Methods:**

Through integrative systems pharmacology approaches, we systematically investigated Shuanghuanglian and its phytochemical constituents against IB, employing multi-omics analysis, ensemble machine learning, and all-atom molecular dynamics (MD) simulations. Network pharmacology revealed 65 target genes associated with Shuanghuanglian’s primary bioactive components (quercetin, kaempferol, wogonin, and luteolin), exhibiting high network centrality.

**Results:**

Using the TCMSP database, we found 65 target genes associated with key active components, such as quercetin and kaempferol, which exhibited strong connectivity in our network analysis. The GeneCards database also identified 40 common target genes shared by Shuanghuanglian and IB. Importantly, BCL2 and IL6 were recognized as key targets in the protein–protein interaction (PPI) network analysis, highlighting their roles in apoptosis and inflammation. Furthermore, analyses using Gene Ontology (GO) and the Kyoto Encyclopedia of Genes and Genomes (KEGG) pathways revealed significant roles in regulating the cell cycle and inflammatory responses. Machine learning techniques identified BCL2 and IL6 as critical genes for therapeutic intervention, supported by molecular docking results that showed strong binding energies. Furthermore, molecular dynamics simulations confirm the stability of the complexes, underscoring the importance of these interactions for treatment efficacy.

**Conclusion:**

We used a variety of analytical methods, and finally identified the potential active ingredients of Shuanghuanglian as kaempferol, quercetin, wogonin, and luteolin. The active ingredients target BCL2 and IL6 and play a therapeutic role in avian infectious bronchitis by inhibiting apoptosis and reducing inflammatory response.

## 1 Introduction

Infectious Bronchitis (IB) is a highly contagious viral disease caused by the Infectious Bronchitis Virus (IBV), which significantly impacts poultry production worldwide. This disease primarily targets the respiratory system of chickens, resulting in severe economic losses due to increased mortality rates, poor weight gain, and decreased egg production (1–3). Although vaccination is commonly employed, the emergence of various IBV serotypes and strains complicates effective immunization efforts, presenting a substantial challenge for poultry health management (4–6).

Current control strategies mainly rely on vaccination and supportive care; however, these approaches often face limitations, including low vaccine efficacy and the slow development of antiviral drugs (7, 8). Recent research has pointed to the potential of traditional Chinese medicine (TCM) in addressing complex diseases, including IB. As a traditional Chinese herbal medicine, Shuanghuanglian has the advantages of low side effects and low drug resistance. Moreover, Shuanghuanglian has the effects of dispelling wind and relieving exterior, clearing heat and detoxifying, and is used for colds caused by exogenous wind-heat (9). At the same time, the clinical symptoms of avian infectious bronchitis include cough, respiratory inflammation, dyspnea, etc. (10), so Shuanghuanglian can be treated symptomatically. Shuanghuanglian is composed of Honeysuckle flower, *S. baicalensis*, and *Forsythia suspensa*. Honeysuckle flower has anti-inflammatory effects and protects the cardiovascular system (11). *S. baicalensis* is determined to have antioxidant, anti-inflammatory, immunomodulatory, anti-apoptotic, anti-cancer and anti-viral effects (12). *Forsythia suspensa* can be used to treat fever, inflammation, gonorrhea, carbuncle and erysipelas (13). Among the options in Traditional Chinese Medicine (TCM), the compound Shuanghuanglian shows promise in preliminary studies (14, 15). However, there is still a major gap in understanding how these herbal components exert their therapeutic effects on IBV and its related pathways. This gap highlights the need for in-depth investigation and underscores the importance of integrating modern scientific methodologies with Traditional Chinese Medicine to develop effective interventions against IB. (16, 17) This study takes a multi-faceted approach that combines pharmacology from Traditional Chinese Medicine with modern bioinformatics techniques, such as network pharmacology, molecular docking, and molecular dynamics simulations.

Network pharmacology is a new field based on systems biology, the analysis of biological networks, and the identification of specific signaling nodes to create multi-target drug molecules. Network pharmacology systematically studies the relationships among key factors such as drugs, diseases, and targets, thereby aiming to reduce adverse drug reactions and improve the success rate of new drug development.

This integrative strategy aims to provide a comprehensive exploration of the active ingredients in Shuanghuanglian and their interactions with key pathological targets in IBV infection. To achieve this, the study employs advanced computational methods to identify both the therapeutic potential of these ingredients and their mechanisms of action against IBV (18).

## 2 Materials and methods

### 2.1 Screening of active components and related targets of traditional Chinese medicine

We conducted a thorough search in the Traditional Chinese Medicine System Pharmacology Database and Analysis Platform [TCMSP (19)]. We aimed to identify the chemical components of traditional Chinese medicinal materials by entering the keywords “Honeysuckle flower,” “*S. baicalensis*,” and “*Forsythia suspensa*.” To enhance the accuracy and relevance of our findings, we applied specific screening criteria. Specifically, we set the criteria for drug-likeness (DL) to be ≥0.18 and oral bioavailability (OB) to be ≥30% (20, 21). Through this meticulous screening process, redundant components were effectively filtered out. Following this, we identified the active components of honeysuckle, *S. baicalensis*, and forsythia, along with their corresponding target genes. Next, we searched the UniProt protein database (22) for verified chicken genes using the query “*Gallus gallus*.” Subsequently, we downloaded the screened results, imported them into the R language with the active components obtained by TCMSP, and used the package for matching and screening. The target genes associated with the active components of Shuanghuanglian were then transformed into standardized gene names (23).

### 2.2 Construction of traditional Chinese medicine-target network

The active components were, respectively, categorized as Honeysuckle flower-JYH, *S. baicalensis*-HQ, and *Forsythia suspensa*-LQ. After importing the active components and their target gene proteins into Cytoscape 3.9.1, we systematically constructed the Traditional Chinese Medicine-target interaction network. Through an in-depth network analysis, we identified the potentially crucial active components of Shuanghuanglian (24, 25).

### 2.3 Acquisition of Targets Related to chicken infectious bronchitis and screening of common targets

We searched the GeneCards database (26) for “Infectious Bronchitis” to identify target genes relevant to chicken infectious bronchitis. Moreover, we imported the target genes of chicken infectious bronchitis and Shuanghuanglian into a Venn diagram website,^1^ which allowed us to identify the intersection and reveal the common target genes shared by both.

### 2.4 Construction of Shuanghuanglian-common target network

We utilized Cytoscape 3.9.1 (27) to generate an interaction network diagram of the active components of Shuanghuanglian and their associated target genes linked to chicken infectious bronchitis (28–30).

### 2.5 Protein–protein interaction (PPI) network analysis and plotting

We submitted the shared target genes of Shuanghuanglian and chicken infectious bronchitis to the STRING protein interaction database for analysis (31). We chose “*Gallus gallus*” for the analysis, set the lowest interaction score to 0.4, and then exported the results. Then we used the built-in plug-in CentiScaPe 2.2 for subsequent analysis. We identified the target genes based on three topological parameters: betweenness, closeness, and degree. The sorted data, arranged in descending order by degree values, resulted in the identification of potential core targets (32).

### 2.6 GO function and KEGG pathway enrichment analysis

The common target genes were imported into the biological information database DAVID (33). Next, we selected the “Functional Annotation Tool” function. Following the website instructions, the sequence attribute “OFFICIAL_GENE_SYMBOL” was chosen, and the species was set to “*Gallus gallus*, “then the information was submitted to begin analysis. We then submitted the information for analysis, and upon completion, we obtained data on biological processes (BP), cellular components (CC), molecular functions (MF), and KEGG pathways. We filtered the results to exclude data with *p* values ≥0.05. We then imported the processed data, along with their corresponding p values and counts, into the micro-bioinformatics website^2^ for visualization. This produced charts for GO function and KEGG pathway enrichment analysis.

### 2.7 Machine learning

We optimized the selection of target gene proteins by analyzing the core proteins identified in section 1.3 and retrieving gene samples from the E-TABM-1128 dataset in the ArrayExpress database. We used three machine learning methods: LASSO, Support Vector Machine Recursive Feature Elimination (SVM-RFE), and Random Forest (RF) (34) to evaluate the key genes’ importance in predicting sample groupings. LASSO (35) which stands for Least Absolute Shrinkage and Selection Operator, is a linear regression technique that reduces the coefficients of unimportant features to zero, effectively selecting the most significant. Using the glmnet package, we determined the optimal regularization parameter, lambda, through 5-fold cross-validation. At this optimal lambda value, LASSO effectively selected genes that had a significant impact. The SVM-RFE algorithm aims to distinguish different types of samples by finding an optimal hyperplane. We conducted Support Vector Machine Recursive Feature Elimination (SVM-RFE) using the e1071 and sigFeature packages. This process involved five-fold cross-validation to eliminate features with lower contributions and to determine the optimal number of features based on error rate curves. The Random Forest method ranked key genes for feature expression according to their Gini index. This ranking helped us to identify the most important genes for analysis. The main advantages of the random forest algorithm include high accuracy and robustness, the ability to effectively avoid overfitting, the ability to process high-dimensional data, and the ability to evaluate the importance of features. These three algorithms often appear in the existing research. It can be seen that the effect is good and the application is wide. After the three machine learning algorithms converged, we identified the overlapping genes as significant components of our analysis.

### 2.8 Molecular docking

To verify the binding of target gene proteins to the active components of Shuanghuanglian, we downloaded their 3D structures from the TCMSP database and saved them in mol2 format. We prepared the structures using AutodockTools 1.5.7 (36) by removing water, adding hydrogen, and selecting torsion bonds. We designated these structures as small molecule ligands. We downloaded the 3D structures of target proteins from the UniProt database and saved them in PDB format. Then, we imported these structures into AutoDockTools version 1.5.7 for processing, which involved removing water, adding hydrogen, and calculating charges. The proteins were designated as macromolecular receptors. We used the Autodock Vina plugin (37) to dock the macromolecular receptors with small molecule ligands. We configured the docking box to cover the entire protein structure and subsequently modified the docking command parameters based on this setup before executing the docking command. We exported the molecular binding energy results as PDBQT format files and visualized them using PyMOL software.

### 2.9 Molecular dynamics simulation

We utilized Gromacs 2023 (38) for molecular dynamics simulations to analyze and confirm the strength and stability of receptor-ligand interactions. We first processed the small molecule ligand with Sobtop to create its topology, and then Gromacs generated the topology for the macromolecular receptor before the simulation. We employed the GAFF (39) force field and SPC216 water model to create a physiological environment for the simulation. The GAFF force field is simple and has good accuracy. It is suitable for dealing with protein-small molecule systems, and it is a comprehensive force field that almost covers the organic chemical space composed of C, N, O, S, P, H, F, Cl, Br, and I. The simulation box was defined as a dodecahedron, ensuring that the complex was at least 0.1 nm from the box edge. Since there was no net charge in the simulation system, ions were added to the system. During the simulation, we applied the steepest descent method for energy minimization over 50,000 steps. The system was then equilibrated using NVT and NPT, with a time step of 2 fs and a total duration of 100 ps to stabilize the system. Finally, we executed a 100 ns MD simulation at 2 fs intervals. After completion, we used Gromacs’ built-in commands to analyze the results, including RMSD, the nuber of hydrogen bonds, the radius of gyration and total energy. The data were then exported and visualized using QTGrace v0.26 and Pymol software.

## 3 Results

### 3.1 Screening of active components and related targets of Shuanghuanglian

**Figure 1:**
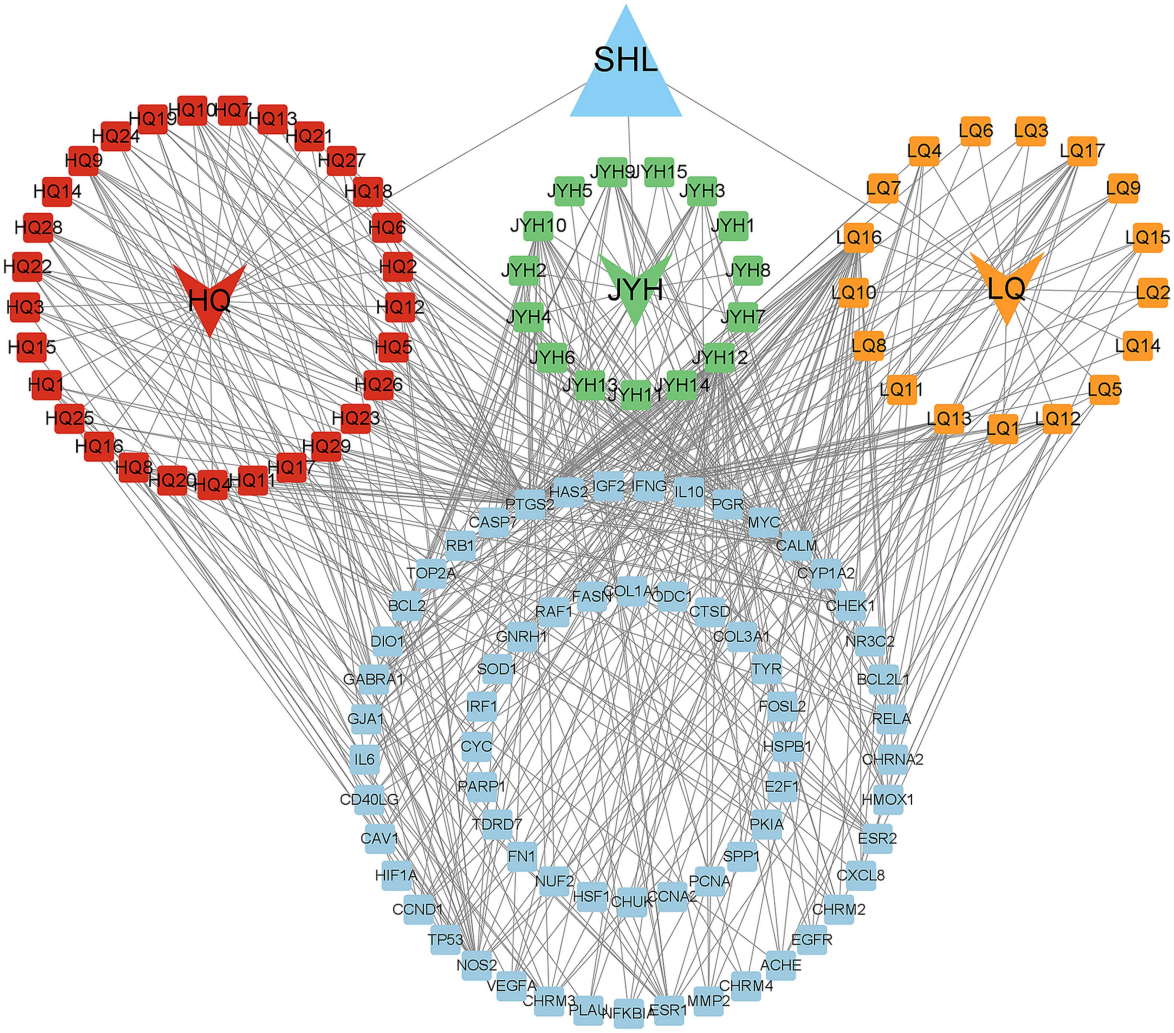
Active ingredients of Shuanghuanglian and the gene network diagram of Chicken Infectious Bronchitis target genes. The red node represents the potential active ingredient of *S. baicalensis*, the green node represents the potential active ingredient of Honeysuckle flower, the yellow node represents the potential active ingredient of *Forsythia suspensa*, and the blue rectangular node represents 65 target proteins.

This study identified the three primary components of Shuanghuanglian using the TCMSP database. We applied the criteria of OB ≥ 30% and DL ≥ 0.18. We identified a total of 15 active ingredients in Honeysuckle flower, 29 in *S. baicalensis*, and 17 in *Forsythia suspensa* (Supplementary Table 1). By screening the active ingredients, we set the stage for future research. To further clarify the action targets of these active ingredients, we utilized the UniProt database to standardize the identification of target genes associated with the efficacy of the active ingredients (Supplementary Table 2). We constructed a network diagram illustrating the target genes of Shuanghuanglian (Figure 1; Supplementary Tables 3, 4). The network diagram illustrates the intricate relationships between 65 target gene proteins and their active components. We ranked the potential active components of Shuanghuanglian based on their degree values obtained from the topological analysis of the network diagram. The four active components with the highest degree values were quercetin, luteolin, kaempferol, and wogonin. These four active components connect to multiple target genes in the network, suggesting they may be crucial for the pharmacological effects of Shuanghuanglian. This finding offers valuable insights for further research into the mechanisms of Shuanghuanglian’s action and establishes a foundation for future molecular docking and dynamics simulations.

### 3.2 Screening of common targets of infectious bronchitis in chickens and active components of Shuanghuanglian

**Figure 2:**
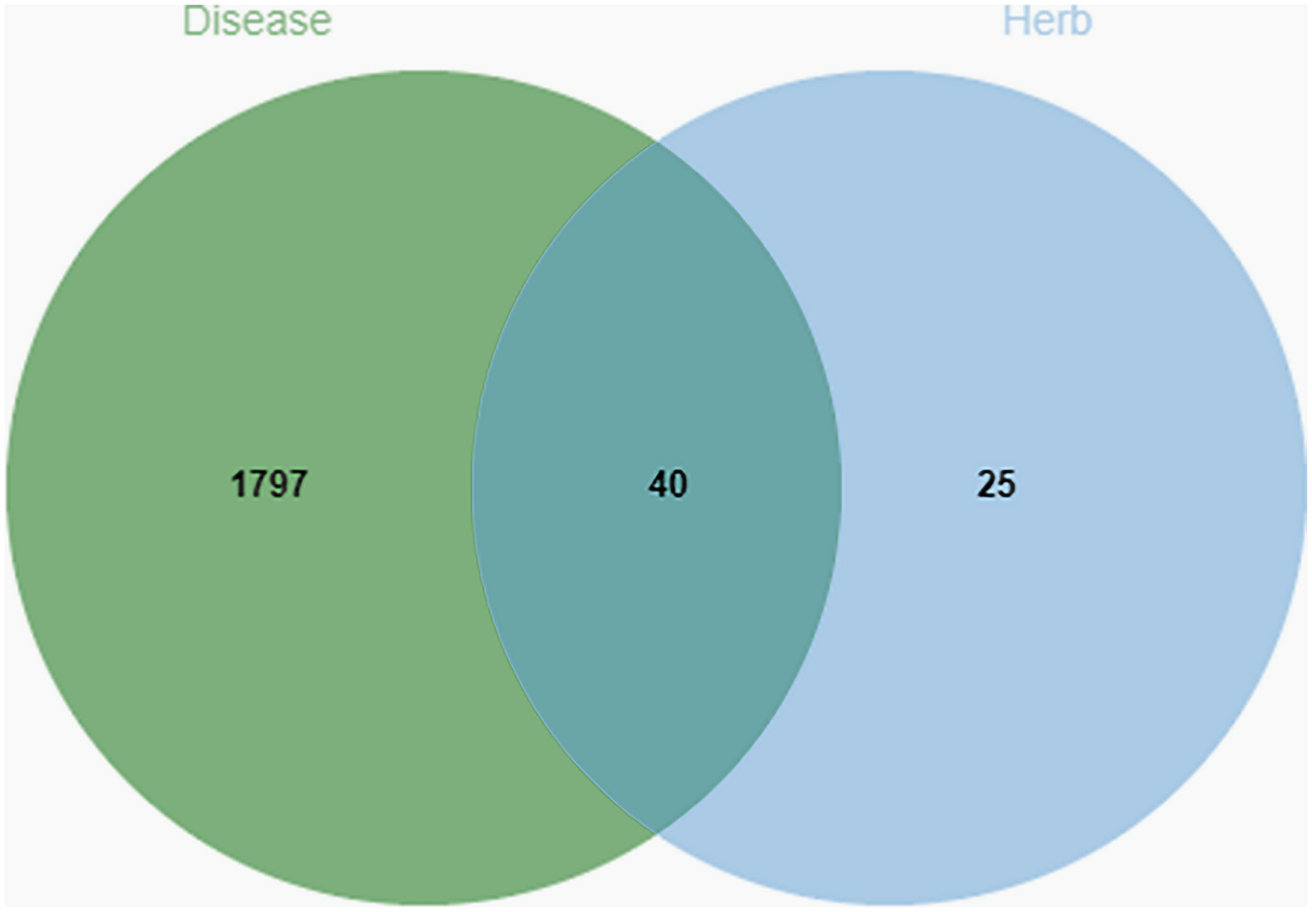
Venn diagram of chicken infectious bronchitis targets and Shuanghuanglian active ingredients. The green part is the target of the disease, the blue part is the target of Shuanghuanglian, and the intersection part is the common target of the disease and the drug.

Our goal was to examine how Shuanghuanglian affects infectious bronchitis in chickens by identifying 1,837 related target genes from the GeneCard database. Next, we compared the disease-related target genes with the 65 target genes identified earlier from the active components of Shuanghuanglian. Using a bioinformatics visualization tool, we created a diagram to show the overlap between the target genes linked to both the disease and Shuanghuanglian’s active components (Figure 2). Our analysis revealed that 40 target genes were common to both the disease-related genes and the active components of Shuanghuanglian. The identification of these 40 shared target genes provides valuable insights into the potential therapeutic mechanisms of Shuanghuanglian in treating infectious bronchitis in chickens. These common targets may represent key molecules through which Shuanghuanglian exerts its therapeutic effects and suggest avenues for further functional validation and research into the mechanisms of drug action. Additionally, this finding highlights the potential of Shuanghuanglian as a multi-target compound for treating complex diseases.

### 3.3 Protein–protein interaction (PPI) network analysis of infectious bronchitis in chickens

**Figure 3:**
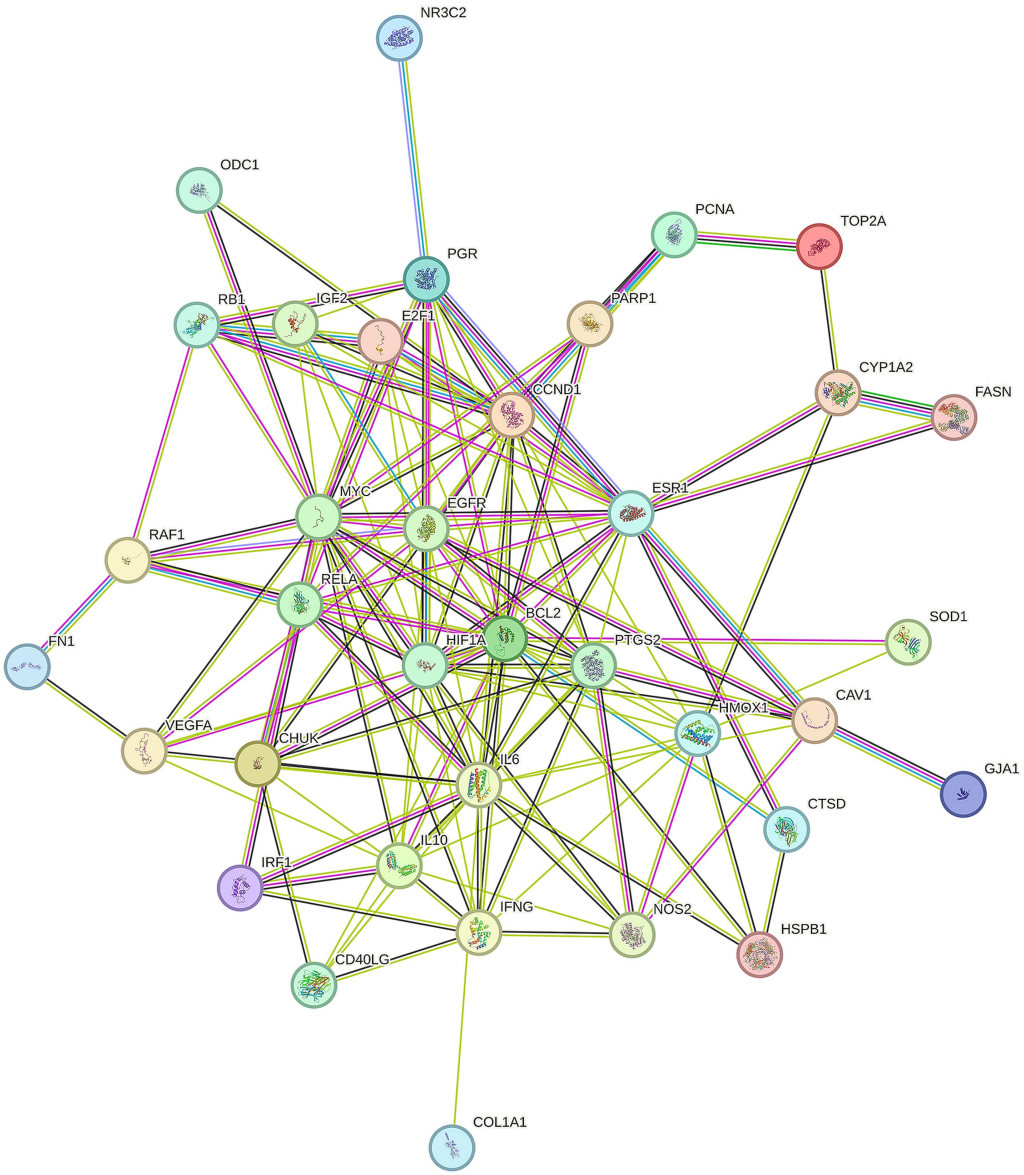
Chicken Infectious Bronchitis protein–protein interaction map. There is a helical structure inside some nodes in the figure, which indicates that the three-dimensional structure of the protein is known. If unknown, the interior of the node is empty. The line between nodes represents the interaction between two proteins, and multiple lines represent multiple interactions.

**Figure 4:**
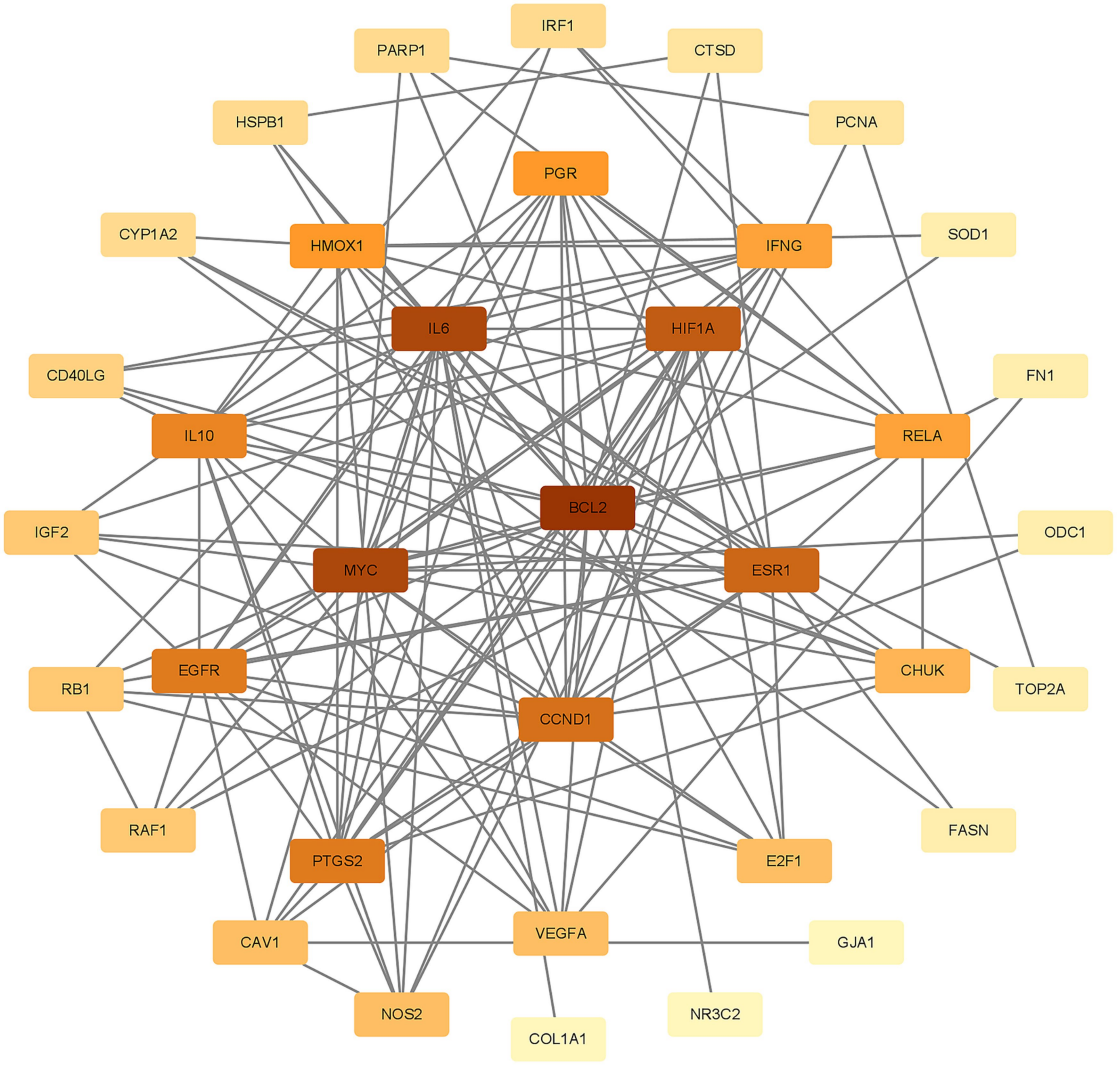
Chicken Infectious Bronchitis Protein–Protein Interaction Network. The color depth of the nodes in the graph is arranged according to the degree value, and the darker the color, the greater the degree value.

**Figure 5:**
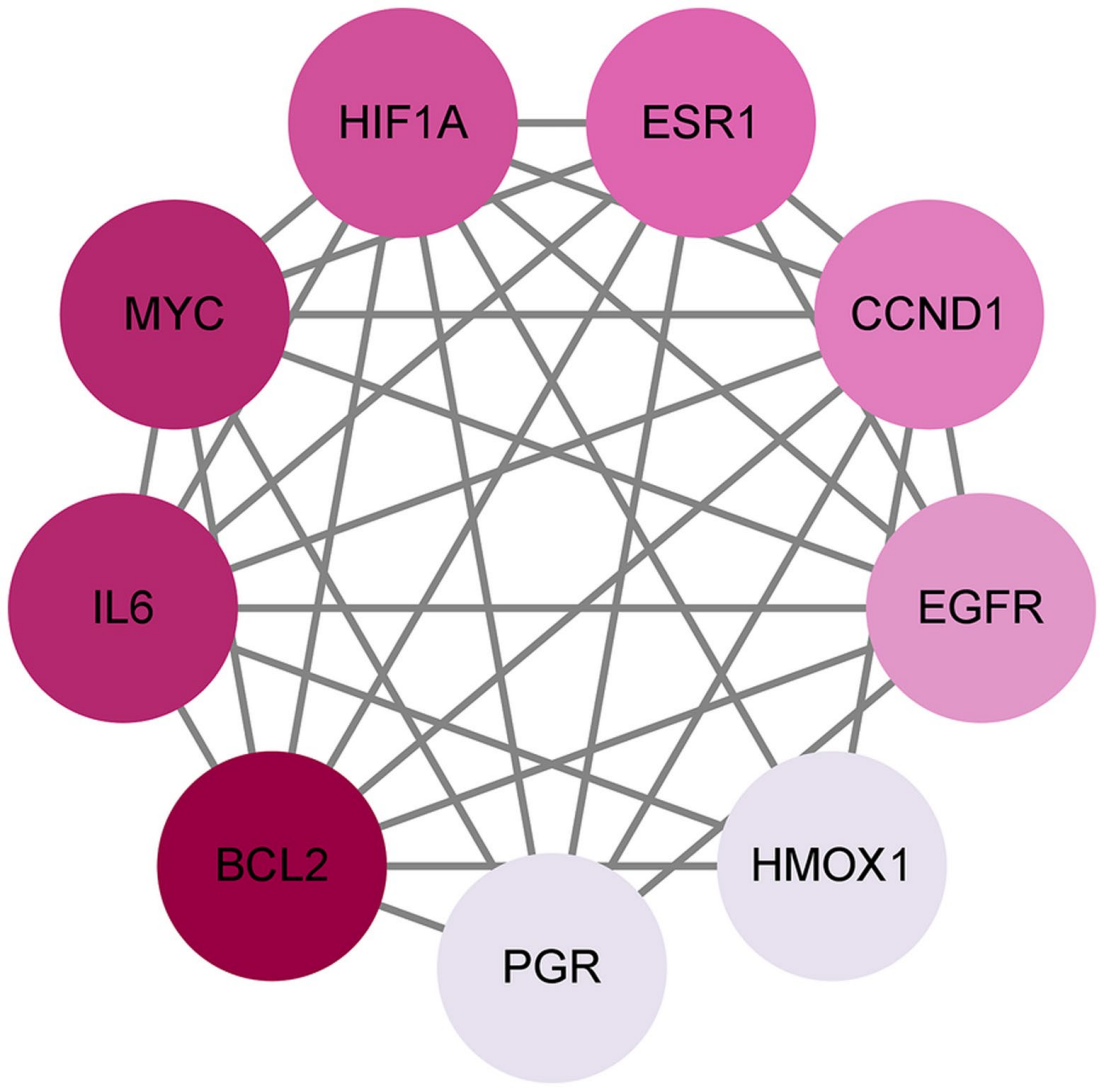
Potential core targets of avian infectious bronchitis. The color depth of the nodes in the graph is arranged according to the degree value, and the darker the color, the greater the degree value.

To better understand how Shuanghuanglian combats infectious bronchitis in chickens, we performed a PPI network analysis targeting 40 previously identified common proteins. We first created a PPI network using the String database, as shown in Figure 3. After optimizing the data by removing unconnected proteins and setting a minimum interaction score of 0.4, we identified 36 relevant proteins. Next, we visualized the 36 proteins with Cytoscape, producing a clearer protein–protein interaction network diagram (Figure 4; Supplementary Table 5). Further analysis allowed us to identify nine potential core target proteins, which are detailed in Figure 5. The nine core targets and their main functions are as follows: 1. BCL2: related to the inhibition of apoptosis; 2. IL6: involved in inflammatory responses; 3. MYC: regulates cell growth, division, and apoptosis; 4. HIF1A: acts as a hypoxia-inducible factor; 5. ESR1: estrogen receptor; 6. CCND1: a cell cycle protein; 7. EGFR: plays an important regulatory role in cellular physiological processes; 8. HMOX1: involved in the cellular response to oxidative stress; 9. PGR: progesterone receptor gene. By identifying these core targets, we gain crucial insights into the molecular mechanisms of Shuanghuanglian’s treatment of infectious bronchitis in chickens, paving the way for future experimental validation and drug development. This finding suggests that Shuanghuanglian could have therapeutic effects by impacting biological processes like apoptosis, inflammation, the cell cycle, and oxidative stress. This reflects the multi-target and multi-pathway action characteristics typical of traditional Chinese medicine compounds.

### 3.4 Gene ontology (GO) functional enrichment analysis

**Figure 6:**
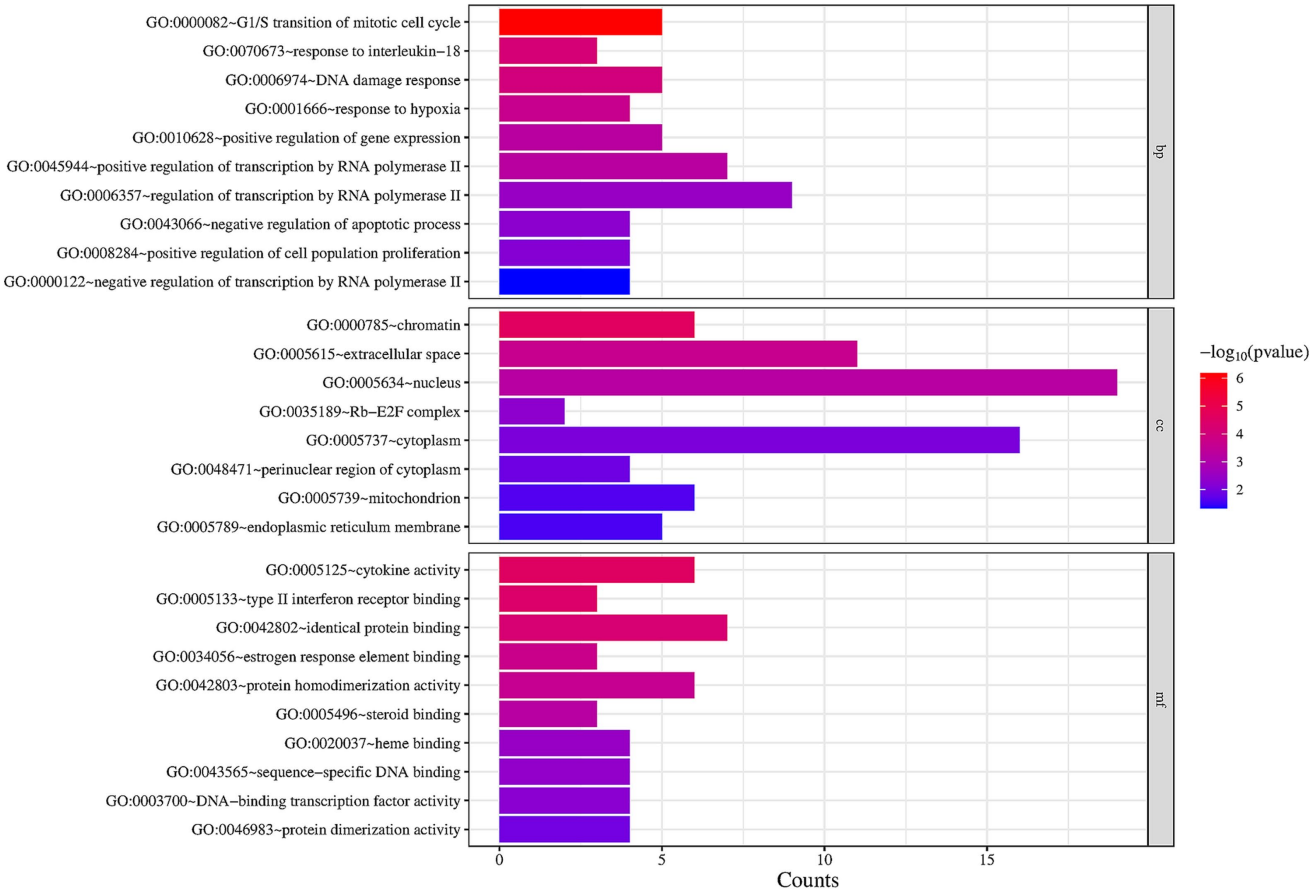
GO enrichment analysis diagram. The darker the color is, the higher the significance is.

We performed a GO functional enrichment analysis on 36 previously identified target gene proteins to better understand how Shuanghuanglian treats infectious bronchitis in chickens. Utilizing the David database, we identified 28 significantly enriched GO entries, which encompassed 10 BP, 8 CC, and 10 MF. These findings were visualized through a micro-bioinformatics mapping platform (Figure 6; Supplementary Table 6). The analysis emphasizes the key biological processes, cellular components, and molecular functions that Shuanghuanglian may interact with when treating infectious bronchitis in chickens. The enrichment analysis shows that Shuanghuanglian mainly affects biological processes, including mitosis regulation and response to interleukin-18. This indicates that Shuanghuanglian likely influences disease progression by affecting both cell proliferation and gene expression. The analysis identifies cellular components such as the nucleus and chromatin, suggesting that Shuanghuanglian’s actions are likely localized within these organelles, impacting gene expression and cellular functions. In terms of molecular functions, the enrichment analysis underscores the significance of cytokine activity, which is closely associated with inflammatory responses and immune regulation, implying that Shuanghuanglian may exert its therapeutic effects by modulating inflammation and immune responses (40). Overall, the GO enrichment analysis results offer valuable insights into how Shuanghuanglian treats infectious bronchitis in chickens, providing a critical theoretical basis for future experimental designs and targeted therapeutic strategies.

### 3.5 KEGG pathway enrichment analysis

**Figure 7:**
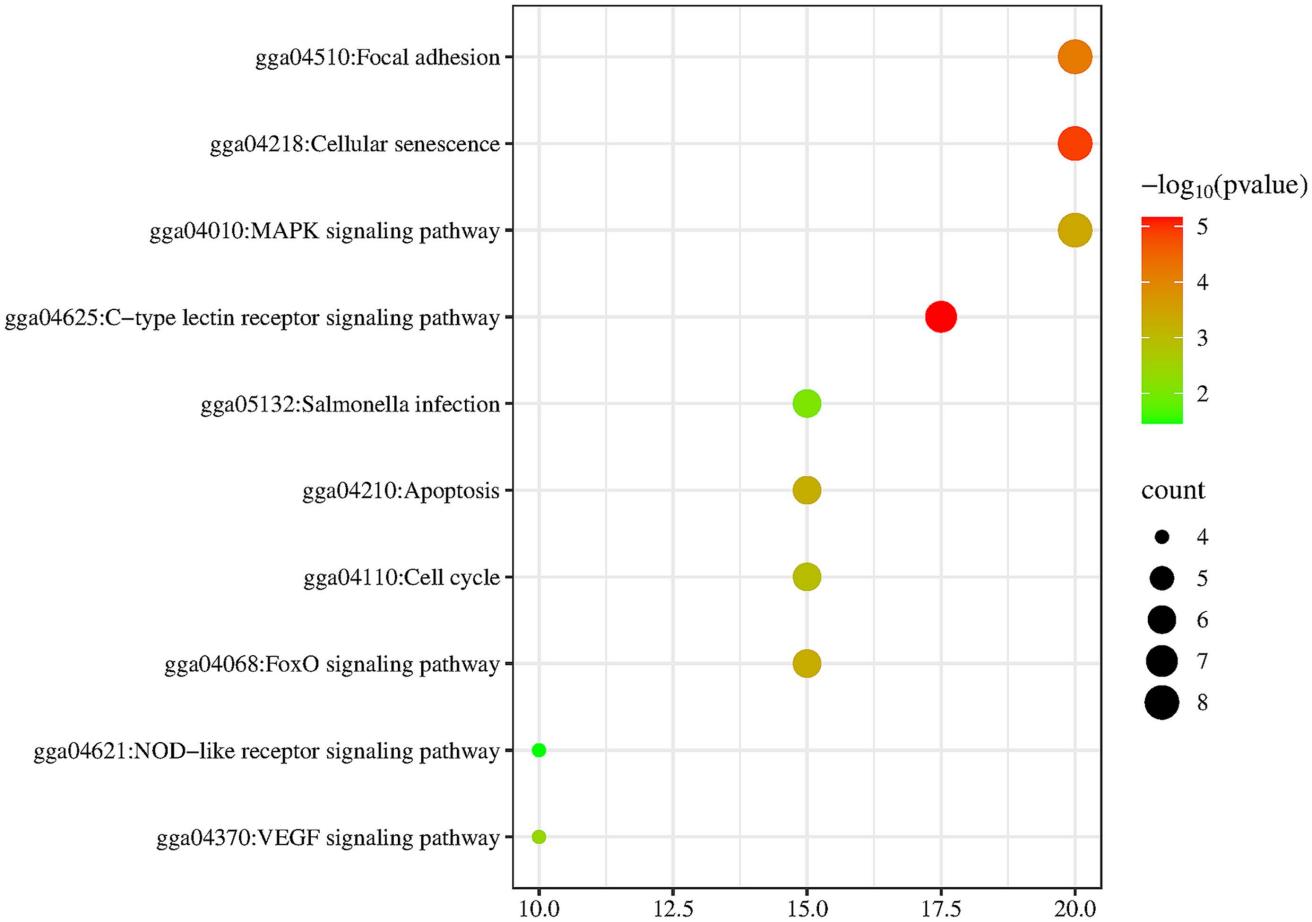
KEGG pathway enrichment analysis diagram. The darker the color is, the higher the significance is.

**Figure 8:**
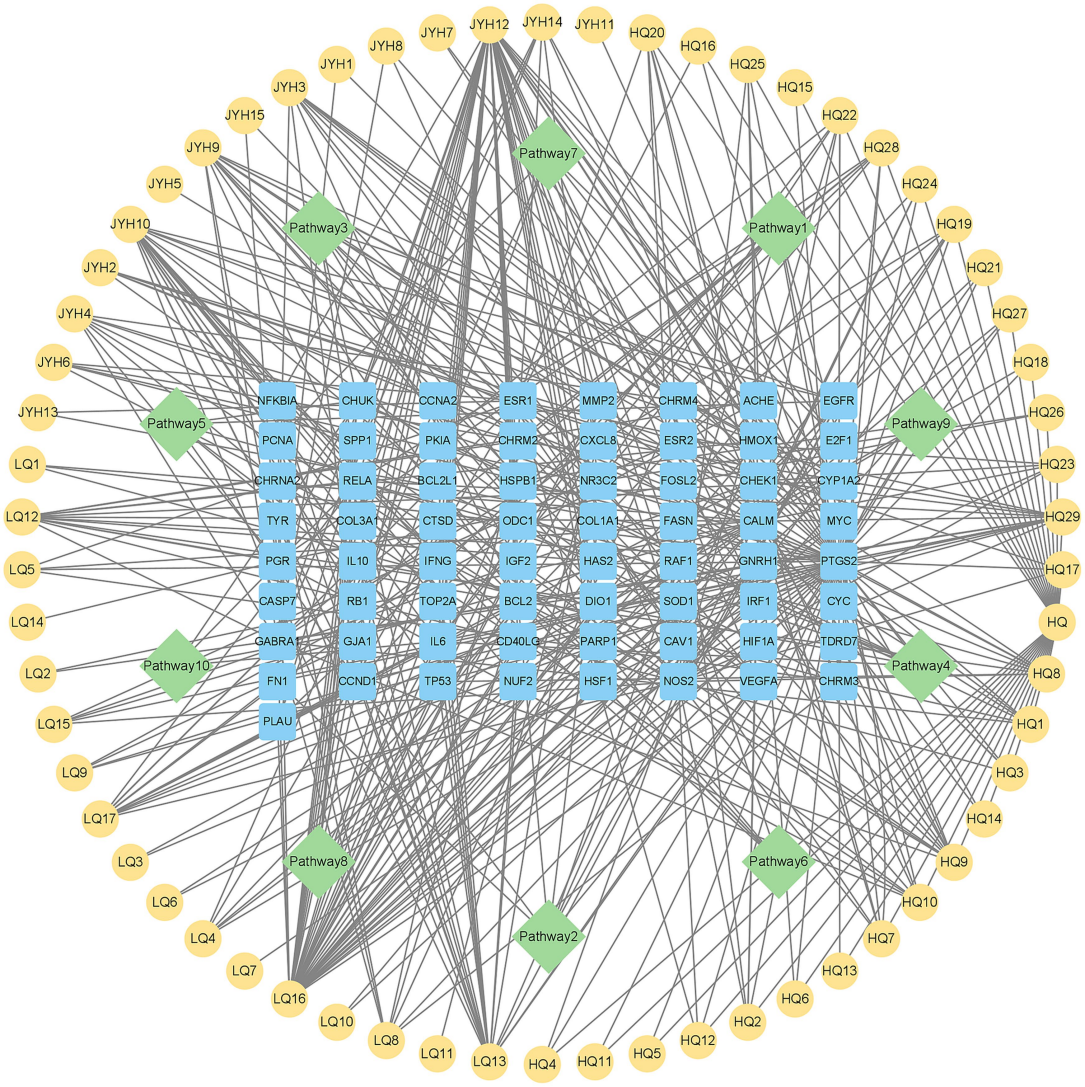
Drug active ingredient-pathway-target gene protein network diagram. The yellow node represents the active ingredient of the drug, the blue node represents the common target of the disease and the drug, and the green node represents 10 kegg pathways.

To clarify how Shuanghuanglian treats infectious bronchitis in chickens, we performed a KEGG pathway enrichment analysis on the shared targets between Shuanghuanglian and the disease. We used the DAVID database to identify 10 significantly related signaling pathways. The results were visualized with a micro-bioinformatics mapping platform (Figure 7; Supplementary Table 6). Our analysis indicates that the shared target genes of Shuanghuanglian and infectious bronchitis are primarily associated with key pathways, such as adhesion plaques, cellular senescence, and MAPK signaling. The enrichment of these pathways implies that Shuanghuanglian could exert its therapeutic effects by regulating various processes, including cell adhesion, cell lifespan, and signal transduction. To clearly depict the relationships among the active components of the drug, signaling pathways, and target genes, we created a protein network diagram using Cytoscape software (Figure 8). This visual representation illustrates the multi-target and multi-pathway characteristics of Shuanghuanglian in treating infectious bronchitis in chickens, highlighting potential connections between active components, targets, and pathways. The enrichment of these pathways suggests that Shuanghuanglian may have therapeutic effects. It may do so by modulating several processes, including cell adhesion, cellular lifespan, and signal transduction. Notably, the involvement of the pathway associated with cellular aging suggests that Shuanghuanglian could help slow disease progression by affecting cellular lifespan. Additionally, the enrichment of the MAPK signaling pathway further supports the potential role of Shuanghuanglian in regulating cell proliferation, differentiation, and inflammatory responses. These findings enhance our understanding of how Shuanghuanglian works and provide a crucial theoretical foundation for future experimental validation and targeted drug development.

### 3.6 Machine learning analysis

**Figure 9:**
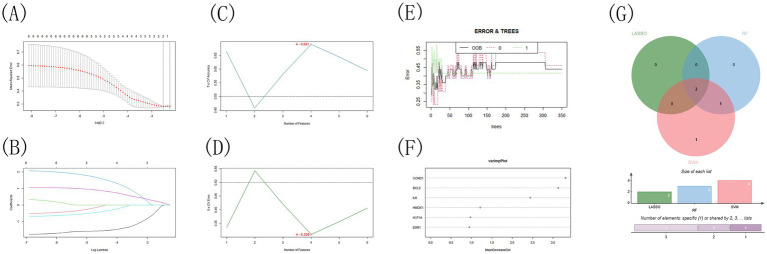
Machine Learning Results Visualization: **(A,B)** The Lasso algorithm is used to verify the gene, **(C,D)** The SVM-RFE algorithm is used to screen the key genes and determine the best combination of genes, **(E,F)** The key genes were screened by random forest algorithm, **(G)** The Venn Diagram of the intersection of three algorithms.

This study aimed to identify key genes involved in the treatment of infectious bronchitis in chickens using Shuanghuanglian by employing three machine learning techniques: LASSO, SVM-RFE, and Random Forest. These methods enhance the reliability of the findings. They also provide thorough validation for identifying the most significant target genes (Supplementary Table 7). The LASSO algorithm analysis identified BCL2 and IL6 as the two most crucial genes. This highlights the importance of BCL2 in apoptosis regulation and IL6 in the inflammatory response during the disease process (Figures 9A,B; Supplementary Table 8). The SVM-RFE method achieved optimal performance by identifying four genes, resulting in the lowest error rate of 0.309 and the highest accuracy of 0.691 (Figures 9C,D; Supplementary Table 9). The four key genes identified are IL6, CCND1, BCL2, and HMOX1. This outcome not only supports the LASSO analysis results but also highlights the significance of cell cycle regulation (CCND1) and the oxidative stress response (HMOX1). Lastly, the Random Forest algorithm evaluated gene importance using the Gini coefficient, identifying three key genes: CCND1, BCL2, and IL6, after establishing a threshold of 2.0 (Figures 9E,F). This finding reinforces the conclusions drawn from earlier analyses, emphasizing the central role of these genes in the disease mechanism. A cross-analysis of results from three methods identified IL6 and BCL2 as core genes (Figure 9G). The consistent identification of these two genes strongly indicates their potential key roles in the treatment of infectious bronchitis in chickens using Shuanghuanglian. IL6 is a significant inflammatory factor, highlighting the need to regulate inflammation during treatment. BCL2, known for its anti-apoptotic properties, indicates that regulating cell survival may be a key mechanism of Shuanghuanglian’s therapeutic effects. These findings provide valuable insights into the mechanism of action of Shuanghuanglian and propose avenues for future experimental validation and the development of targeted therapies. Furthermore, the thorough application of various machine learning methods demonstrates the rigor and comprehensiveness of this study in data analysis.

### 3.7 Molecular docking studies

**Figure 10:**
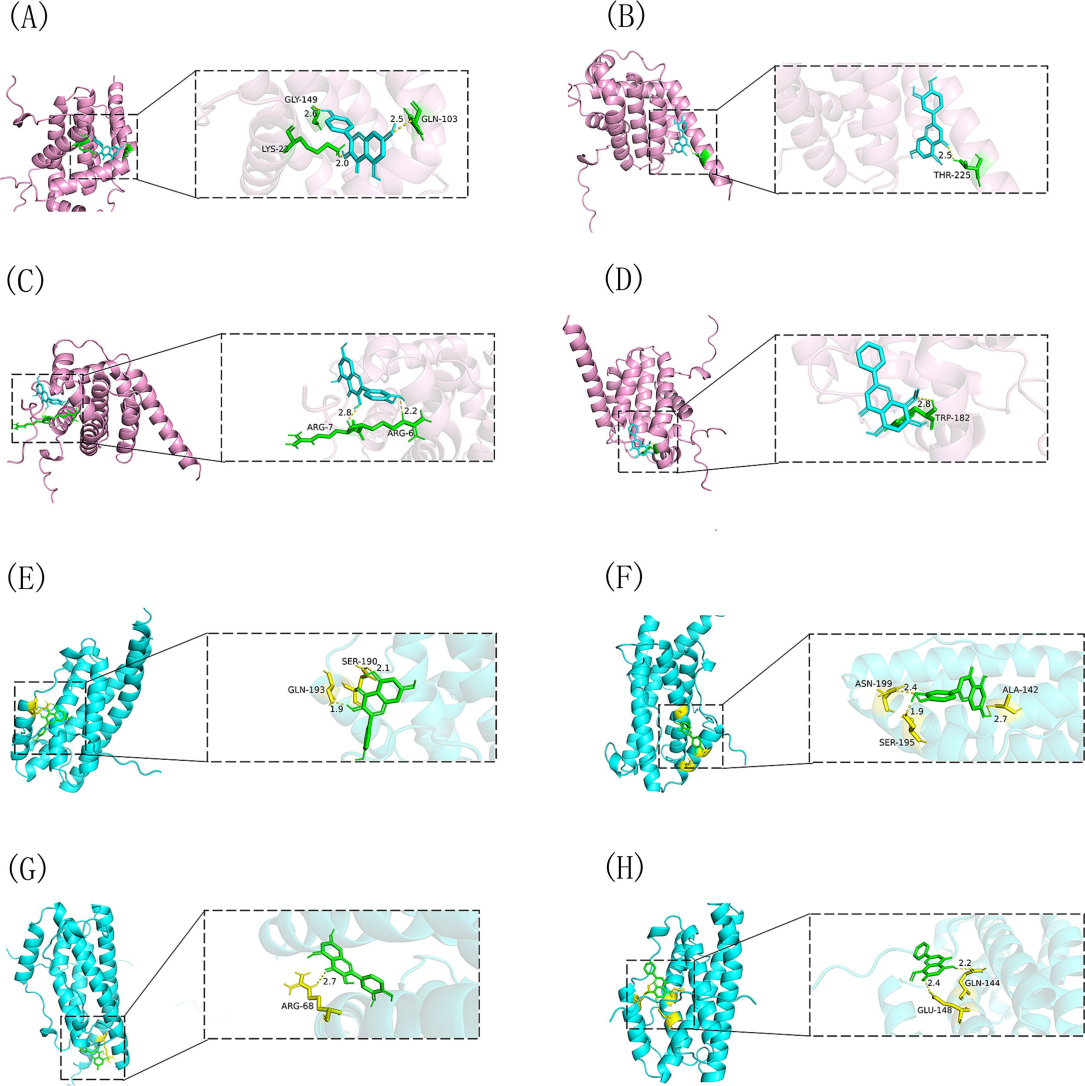
Visual representation of the docking of four drug active components with BCL2 and IL6. **(A)** BCL2-kaempferol, **(B)** BCL2-luteolin, **(C)** BCL2-quercetin, **(D)** BCL2-wogonin, **(E)** IL6-kaempferol, **(F)** IL6-luteolin, **(G)** IL6-quercetin, **(H)** IL6-wogonin.

**Figure 11:**
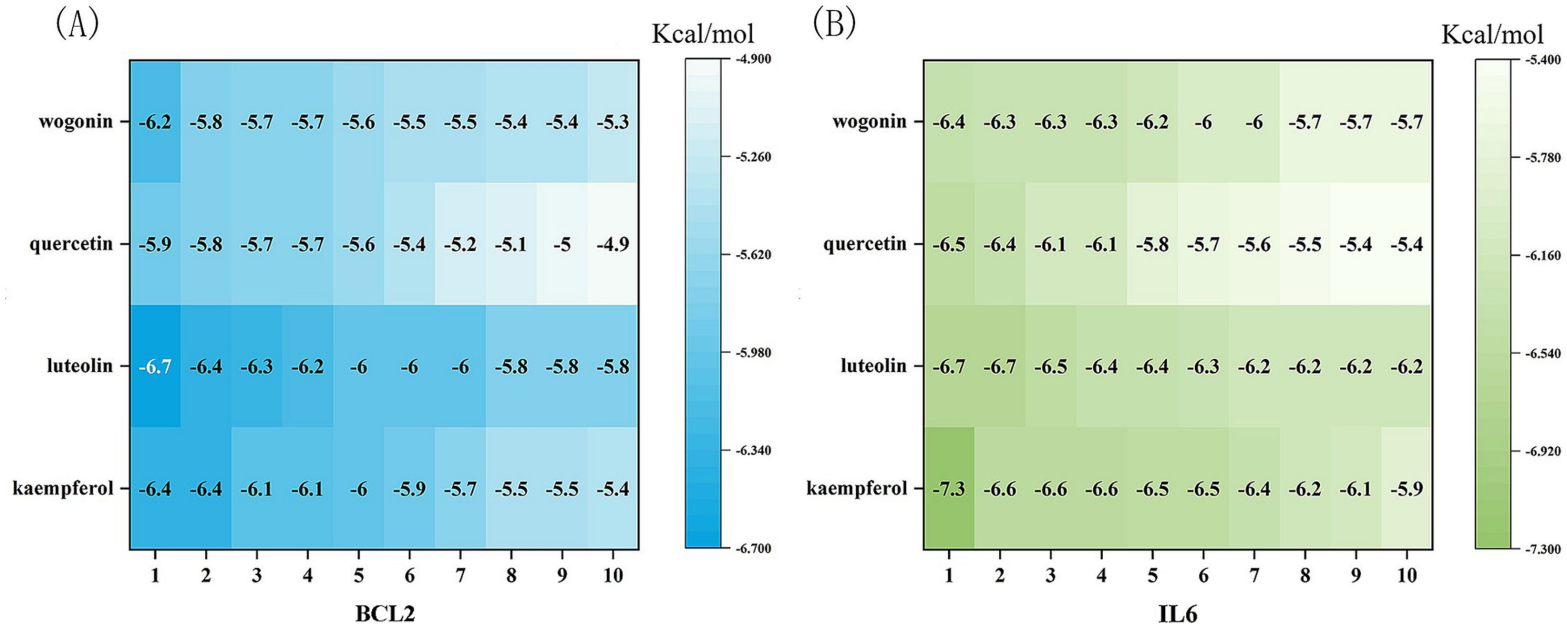
Docking binding energy of four potential active component small molecules with BCL2 and IL6. **(A)** The binding energy heat map of BCL2 with four small molecules, **(B)** The binding energy heat map of IL6 with four small molecules.

We performed molecular docking studies to explore how Shuanghuanglian treats infectious bronchitis in chickens, concentrating on its main active components and their interactions with important target proteins. The selected ligands for this study were kaempferol, luteolin, quercetin, and wogonin. The core target proteins identified through machine learning analysis were BCL2 and IL6. To ensure the reliability of our findings, we performed 10 docking simulations for each receptor-ligand pair. We assessed the results by examining the binding energy, where lower values indicated stronger interactions. From each docking group, we chose the conformation with the lowest binding energy, exported it in PDBQT format, and visualized it in three dimensions using PyMOL software (Figure 10). This visualization clearly shows how the active components bind to the target proteins, offering valuable spatial structural information that improves our understanding of their interactions. To present the docking results more comprehensively, we processed the binding energy data using Origin 2021 software, creating binding energy heatmaps (Figure 11). These heatmaps effectively illustrate the binding strengths of various ligand-receptor combinations, allowing for intuitive comparisons. The analysis reveals that kaempferol, luteolin, quercetin, and wogonin demonstrate strong binding affinities for BCL2 and IL6, with all combinations surpassing the established standard values for binding energy (41). This finding strongly indicates that these active components may exert their therapeutic effects by directly binding to BCL2 and IL6. Specifically, the robust binding with BCL2 suggests that Shuanghuanglian could influence disease progression by regulating the apoptosis process, By inhibiting the transmission of apoptotic signals, it protects cells from programmed death, while the interaction with IL6 points to its potential role in modulating inflammatory responses. It can induce the synthesis of acute phase proteins and promote the aggregation and activation of inflammatory cells. In infectious diseases, IL6 levels are usually elevated, reflecting the severity of the disease. In autoimmune diseases, IL6 may also be abnormally elevated, promoting the development of the disease‌. The molecular docking results not only support the findings from earlier machine learning analyses but also offer direct structural biological evidence explaining how Shuanghuanglian treats infectious bronchitis. Additionally, this analysis indicates that various active components in traditional Chinese medicine might work together, providing new insights into the overall effects of these formulations.

### 3.8 Molecular dynamics analysis

**Figure 12:**
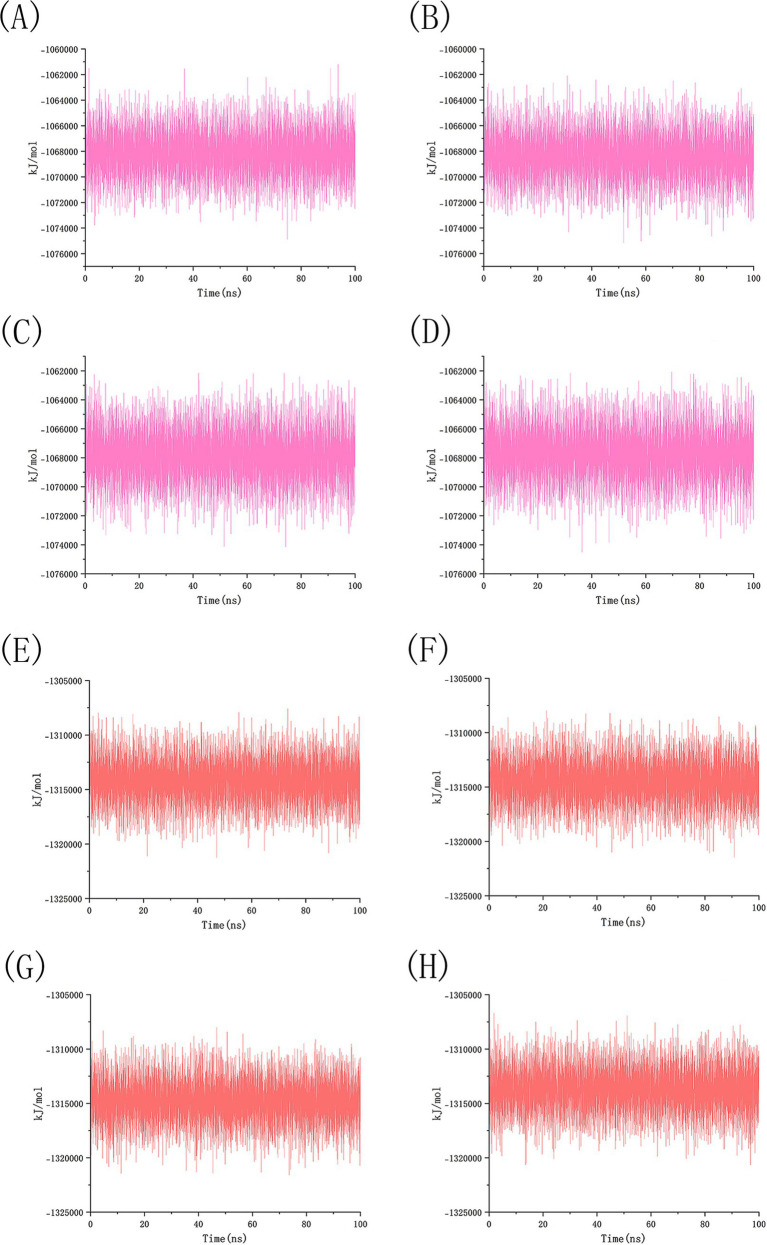
A total energy analysis of BCL2 and IL6 with four potential drug active component small molecules simulation. **(A)** The total energy diagram of BCL2 and kaempferol, **(B)** The total energy diagram of BCL2 and luteolin, **(C)** The total energy diagram of BCL2 and quercetin, **(D)** The total energy diagram of BCL2 and wogonin, **(E)** The total energy diagram of IL6 and kaempferol, **(F)** The total energy diagram of IL6 and luteolin, **(G)** The total energy diagram of IL6 and quercetin, **(H)** The total energy diagram of IL6 and wogonin.

**Figure 13:**
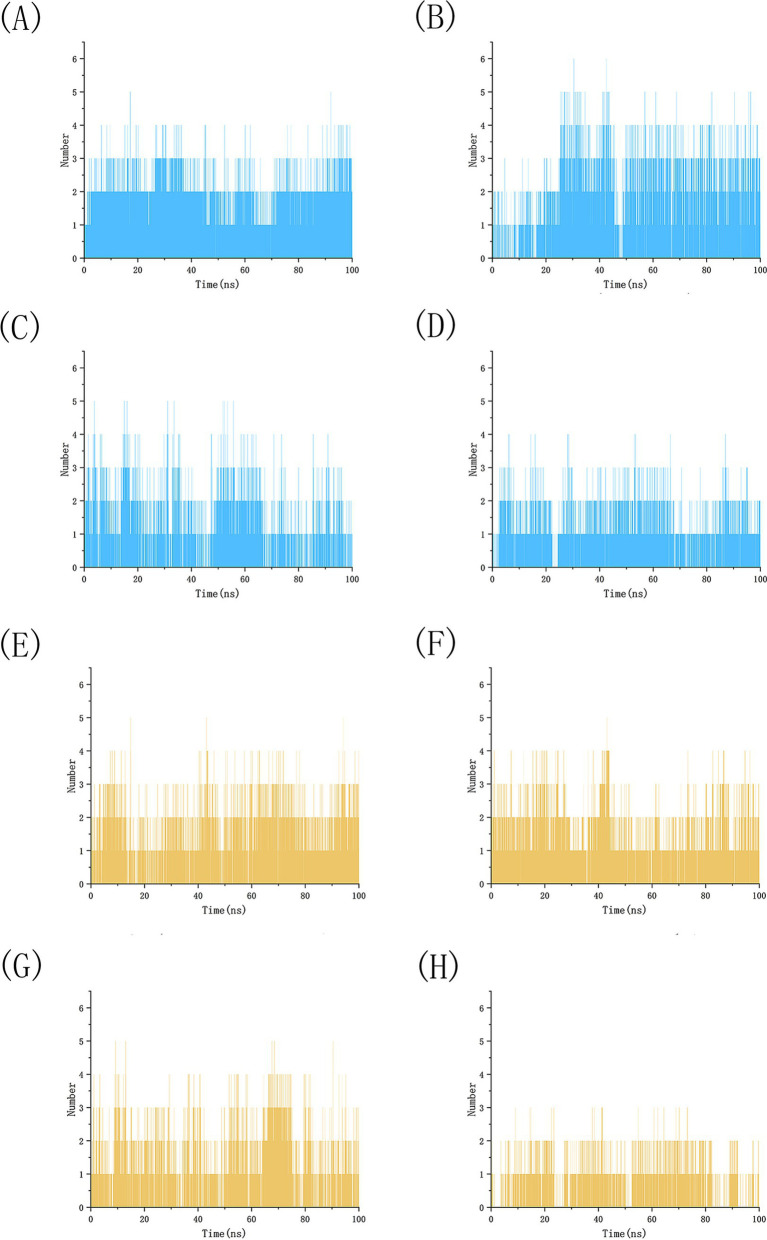
Analysis of the number of simulated hydrogen bonds between BCL2 and IL6 with four potential active drug components. **(A)** BCL2-kaempferol, **(B)** BCL2-luteolin, **(C)** BCL2-quercetin, **(D)** BCL2-wogonin, **(E)** IL6-kaempferol, **(F)** IL6-luteolin, **(G)** IL6-quercetin, **(H)** IL6-wogonin.

**Figure 14:**
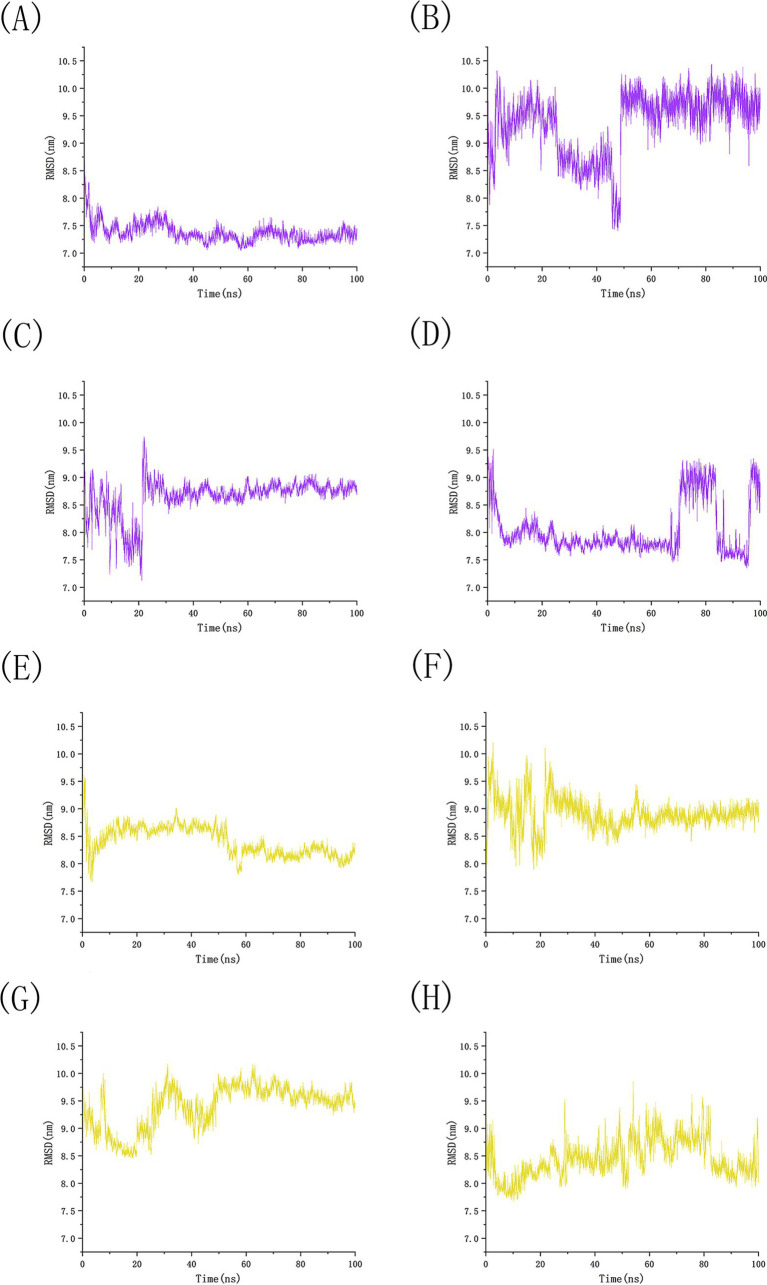
RMSD analysis of potential active component small molecule simulations with BCL2 and IL6. **(A)** The number of hydrogen bonds between BCL2 and kaempferol, **(B)** The number of hydrogen bonds between BCL2 and luteolin, **(C)** The number of hydrogen bonds between BCL2 and quercetin, **(D)** The number of hydrogen bonds between BCL2 and wogonin, **(E)** The number of hydrogen bonds between IL6 and kaempferol, **(F)** The number of hydrogen bonds between IL6 and luteolin, **(G)** The number of hydrogen bonds between IL6 and quercetin, **(H)** The number of hydrogen bonds between IL6 and wogonin.

**Figure 15:**
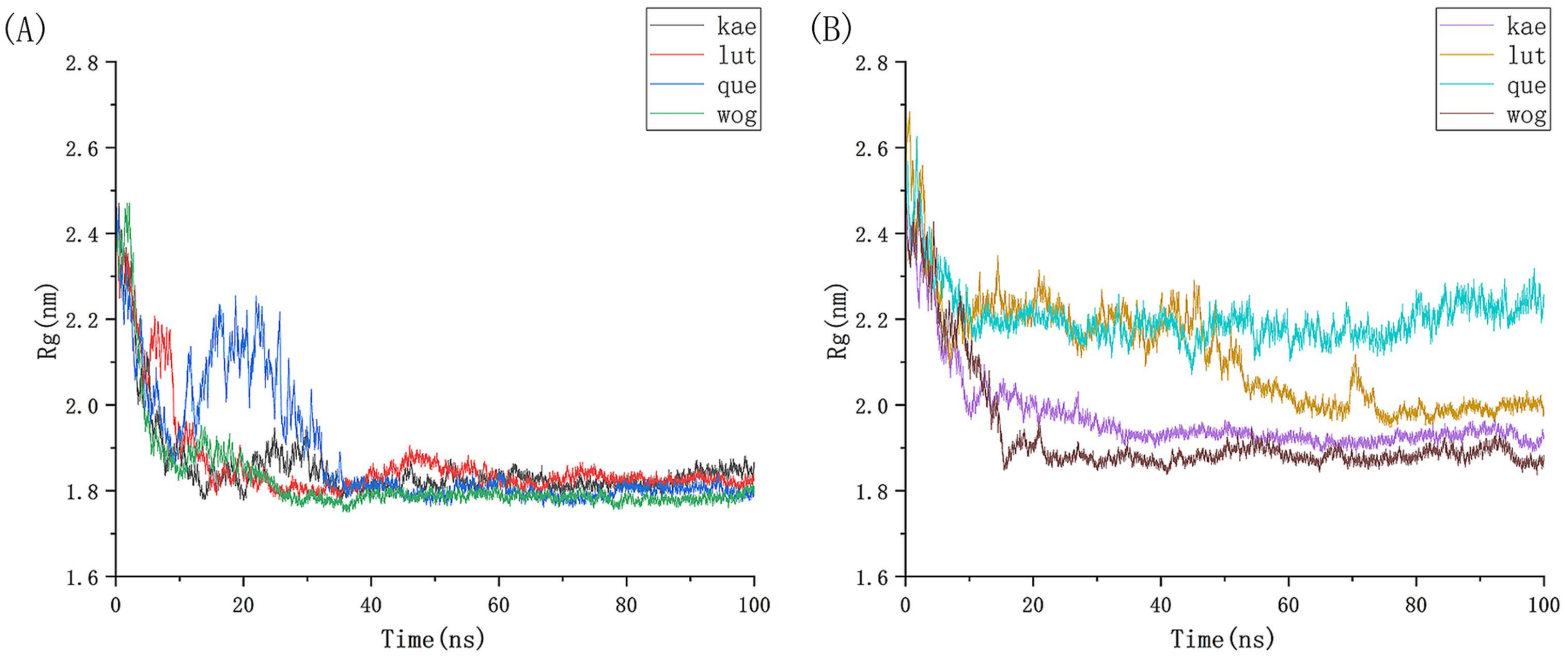
Analysis of the gyration radius of BCL2 and IL6 protein with four potential active drug components in molecular simulation.**(A)** The gyration radius of BCL2 and four small molecules, **(B)** The gyration radius of IL6 and four small molecules.

We conducted 100 ns molecular dynamics simulations on protein-ligand complexes, focusing on total energy, root mean square deviation (RMSD), hydrogen bond counts, and radius of gyration. The total energy reflects the energy conservation state of the simulation system, which is composed of potential energy, kinetic energy and so on. It is an important monitoring index in molecular dynamics simulation. In the simulation process, the change of total energy can help to determine whether the system reaches equilibrium. If the total energy fluctuates abnormally or changes continuously, it may indicate that there are problems such as numerical instability or improper initial conditions in the simulation. Figure 12 shows the molecular dynamics simulation results for BCL2, IL6, and four potential active components. These active components maintain stability with BCL2 between approximately −1,070,000 and −1,065,000 during the 0–100 ns timeframe, and with IL6 between −1,315,000 and −1,310,000, The total energy fluctuations of BCL2, IL6 and the four active components are less than or equal to 5,000, indicating strong binding stability. Figure 13 shows the results of hydrogen bond number analysis; hydrogen bonds are strong intermolecular forces that play a key role in structural stability. Hydrogen bond is a common intermolecular force, which is of great significance in maintaining the spatial structure and biological activity of biological macromolecules. The analysis shows that when binding to BCL2, kaempferol and quercetin exhibit hydrogen bond counts between 0 and 5, wogonin between 0 and 4, and luteolin between 0 and 6. When binding with IL6, kaempferol, quercetin, and luteolin maintain hydrogen bond numbers in the range of 0–5, while wogonin maintains a range of 0–3. These hydrogen bonds play a crucial role in maintaining stability during binding. When the radius of gyration of the molecule is small, the molecule is in a more compact state, and the distance between the atoms inside the molecule is closer, which is easier to form hydrogen bonds. By observing the number of hydrogen bonds, we can study the interaction between proteins and small molecules. Hydrogen bond and RMSD binding analysis showed that hydrogen bonds were generated, indicating that the protein was bound to small molecules. The RMSD of protein atoms is usually used to measure the structural deviation of protein structure before and after simulation. By calculating the deviation degree between the atomic coordinates and the reference conformation, the overall structural stability is evaluated, which is an important index to evaluate the stability of the research system (42). RMSD analysis measures differences in molecular structures by calculating the positional deviations of atoms between two molecules and determining the root mean square of these deviations. RMSD values reflect the amplitude of atomic motion in protein structure resolution, model building, structure alignment, and molecular dynamics simulations. As shown in Figure 14, when binding with BCL2, kaempferol remains stable within the 0–100 ns range, quercetin from 20 to 100 ns, luteolin from 55 to 100 ns, and wogonin from 10 to 60 ns. When binding with IL6, kaempferol remains stable within the 0–100 ns range, quercetin from 60 to 100 ns, luteolin from 25 to 100 ns, and wogonin from 25 to 45 ns. The radius of gyration of a protein can characterize the compactness of molecular conformation, and the size of the rotation radius is influenced by intermolecular interactions, temperature, solvents, etc. The measurement of the radius of gyration can be used for the characterization of protein size and to quantify molecular-scale changes during the denaturation process of proteins (43). The smaller the radius of gyration is, the closer the protein is and the more rigid the structure is, which can help us understand the folding behavior and unfolding behavior of molecules during molecular dynamics simulation. The numerical decrease indicates that the protein is gradually forming a tight folding structure. It shows that it has a strong dense conformation and the value is relatively stable, indicating the stability of the protein structure. Therefore, studying the radius of gyration is crucial for understanding the physical properties and chemical reaction mechanisms of molecules. Figure 15 illustrates that during the 40–100 ns interval, the four active molecules maintain stability between 1.8 and 1.9 nm when bound to BCL2. When binding with IL6, during the 20–100 ns interval, kaempferol and wogonin maintain stability between 1.8–2 nm, quercetin between 2.2–2.3 nm, and luteolin between 1.9–2.1 nm during the 80–100 ns interval. Our research demonstrates that the four molecules interact stably with the BCL2 target protein, significantly strengthening the reliability of molecular docking results.

## 4 Discussion

IB is a highly contagious viral disease affecting chickens, caused by the IBV, which is part of the Coronaviridae family. IB is widespread in commercial poultry farming around the world. It poses significant challenges to the industry, resulting in substantial economic losses from reduced productivity, higher mortality rates, and the costs of control measures (44). Although vaccination strategies have been implemented to manage IB, the emergence of new IBV strains and the limited cross-protection provided by current vaccines hinder effective control of the disease. The complex pathology of IB, combined with the differing immune responses in various chicken breeds, underscores the urgent need for innovative therapeutic strategies beyond traditional vaccination methods.

This study explores the therapeutic potential of Shuanghuanglian, a traditional Chinese medicine, and its active components in treating Infectious Bronchitis. We use system pharmacology and bioinformatics to identify the active ingredients of Shuanghuanglian and their interactions with key target genes related to IB. Our research presents a comprehensive analytical framework that integrates machine learning, molecular docking, and molecular dynamics simulations to demonstrate the multi-target and multi-component effects of Shuanghuanglian against infectious bronchitis. The findings highlight the importance of identifying critical genes involved in the disease mechanism, such as BCL2 and IL6. They also clarify the molecular interactions that may help develop effective therapeutic alternatives for managing infectious bronchitis in poultry farming. The investigation into Shuanghuanglian, a traditional Chinese medicine, as a potential treatment for IB offers a promising alternative to existing therapies. This study highlights the identification of key active components, including quercetin, kaempferol, and luteolin, which have shown notable anti-inflammatory and immunomodulatory properties. These findings suggest that Shuanghuanglian may effectively manage IB through a multi-target approach. Additionally, the research indicates that these active components can interact with essential pathways involved in IB’s development (45).

According to component analysis, the main active ingredient of Honeysuckle flower is Baicalin, the main active ingredient of *S. baicalensis* is Chlorogenic acid, and the main active ingredient of *Forsythia suspensa* is Forsythoside A (23). Among the four potential active ingredients we predicted, wogonin is one of the main active ingredients of *S. baicalensis* (46). Wogonin can inhibit the production of inflammatory cytokines and other inflammatory mediators (47). Luteolin is a typical natural flavonoid in Honeysuckle flower (48) and it has potential anti-inflammatory effects (49). Although quercetin is not the main component of Shuanghuanglian, it has been proved by experiments that it can cooperate with Forsythoside A in *Forsythia suspensa* to produce antioxidant effect (50). Although the content of kaempferol is lower than that of the other three potential active ingredients, the single effect is limited. However, in the process of preparing traditional Chinese medicine prescriptions, the metabolic mixture produced by the oxidation of kaempferol can significantly improve the antioxidant capacity of kaempferol (51).

Through the analysis of network pharmacology and machine learning, we identified two key disease targets BCL2 and IL6, which were consistent with the results of Al-Rasheed et al. (52) and Manswr et al. (53). Al-Rasheed M and Manswr B determined that avian infectious bronchitis was related to IL6. By regulating IL6, it can affect the change of disease. Liu et al. (54) and Chen et al. (55) proved that up-regulation of BCL2 can effectively affect the pathological process of avian infectious bronchitis.

This study suggests that Shuanghuanglian may affect the pathological process of infectious bronchitis through a multi-target regulatory network. In GO analysis, we found that Shuanghuanglian can affect infectious bronchitis in chickens through “G1/S transition of mitotic cell cycle” in biological processes. Shuanghuanglian may inhibit virus-induced excessive apoptosis of host cells and delay cell cycle G1/S transition through interaction with CDK4/cyclin D1 complex (56, 57). This dual effect not only maintains the homeostasis of infected cells, but also destroys the cell cycle microenvironment required for coronavirus replication. Combining the results of biological processes with cell components, we found that Shuanghuanglian can affect the disease by “response to interleukin-18” and “cytokine activity.” The active components of Shuanghuanglian may reduce the nuclear translocation of NF-κB by inhibiting the IL-18R/MyD88 signaling axis, and then down-regulate the overexpression of pro-inflammatory factors such as IL6. This selective regulation of the IL-18/IL6 inflammatory axis is of key significance in alleviating the immunopathological damage of the respiratory tract (58, 59). It is worth noting that the “cellular senescence” in the KEGG pathway reveals a deeper therapeutic mechanism. The cell senescence caused by viral infection forms a vicious cycle through the secretion of inflammatory mediators such as IL6 by SASP, while Shuanghuanglian may enhance the anti-apoptotic ability of senescent cells by up-regulating the expression of BCL2, promote the specific clearance of immune cells, and inhibit the IL6-mediated paracrine aging effect (60). This two-way regulation of aging-inflammation provides a new perspective for improving the function of bronchial epithelial barrier. In addition, the reprogramming of the integrin-FAK signal in the “focal adhesion” pathway may affect the STAT3-dependent cilia repair process by regulating the level of IL6 secretion in the extracellular matrix, suggesting that the temporal and spatial specific regulation of IL6 by Shuanghuanglian may play a differentiated therapeutic role in different pathological stages (61).

The integration of molecular docking and dynamics simulations shows stable interactions between these active components and their targets, indicating a strong binding affinity that could enhance clinical outcomes. The molecular docking analysis indicates that the binding energies of kaempferol, luteolin, quercetin, and wogonin with BCL2 and IL6 are well below the established threshold. This suggests a strong binding affinity that supports their potential effectiveness in treating IB. Among the binding of BCL2, luteolin has the best binding with BCL2, which means that luteolin can inhibit cell apoptosis and increase cell viability by up-regulating BCL2, which is also demonstrated by Xu et al.'s experiment (62). In the binding of IL6, kaempferol has the strongest binding energy, indicating that kaempferol can reduce the inflammatory response by down-regulating the expression level of IL6, thereby reducing inflammation and improving symptoms. In the experiment of Qu et al. (63), it was also found that kaempferol can down-regulate the expression level of IL6.

This finding reinforces the machine learning analysis results, which identified these bioactive components as potential modulators of pathways related to cell apoptosis and inflammation. The strong binding observed in the docking studies bolsters the credibility of the machine learning predictive models. This provides a solid framework for understanding traditional Chinese medicine, particularly the effectiveness of Shuanghuanglian against infectious diseases such as chicken infectious bronchitis. However, we queried the GEO and ArrayExpress databases and searched for data on avian infectious bronchitis through the keywords “IBV” and “Infectious bronchitis.” Unfortunately, there are very few data sets on avian infectious bronchitis, so our analysis results in the machine learning part are not very satisfactory, but we provide a preliminary and useful insight into understanding the model behavior by combining Lasso, support vector machine, and random forest algorithm. We recognize these limitations and commit to working hard to overcome these challenges in future research work to improve the verification effect and reliability of the model.

This study’s molecular dynamics (MD) simulations offer detailed insights into the interactions between active components and the BCL2 and IL6 proteins. Understanding these interactions is crucial for elucidating the mechanisms of potential therapeutic agents. The complex’s stability, shown by total energy fluctuations ranging from −1,070,000 to −1,065,000 during the 100 ns simulation, indicates that the binding is strong and energetically favorable. This stability is vital for drug effectiveness because it shows that the drug can maintain its interaction with the target for a long time, leading to sustained pharmacological effects. Additionally, the hydrogen bond analysis reveals different strengths of interactions among the active components. The variation in hydrogen bonding can significantly influence the selectivity and potency of compounds targeting BCL2 and IL6, ultimately affecting the overall therapeutic efficacy of treatments. The insights gained from these simulations support earlier studies highlighting the critical role of stable protein-ligand interactions in drug design and optimization. The differing binding dynamics of active components like kaempferol, quercetin, wogonin, and luteolin underscore the necessity of evaluating both the quantity and quality of interactions when assessing drug candidates. Differences in hydrogen bond counts may indicate unique structural features and functional groups in each compound, which can affect their pharmacodynamics. For example, the higher hydrogen bond count in luteolin suggests it may interact more flexibly with BCL2 and IL6, which could be linked to increased biological activity. This comparative analysis provides valuable insights for selecting and modifying compounds in drug development, consistent with earlier research that emphasizes the importance of molecular dynamics in predicting binding affinities and optimizing lead compounds. Identifying key genes like BCL2 and IL6 using network pharmacology reveals the complex relationships between immune responses and apoptosis in treating infectious bronchitis.

The findings suggest that focusing on these genes may enhance the therapeutic effects of Shuanghuanglian. This could result in new treatment strategies that integrate the benefits of traditional medicine with modern veterinary practices (64, 65). The poultry industry continues to face challenges due to infectious bronchitis. This study provides valuable insights for developing effective treatment protocols that combine traditional herbal remedies with modern biomedical methods. However, the study has limitations, mainly due to the absence of clinical validation since our research design did not include empirical experiments. This limitation, combined with a relatively small sample size, could affect the generalizability and reliability of our findings. While our findings illuminate potential mechanisms by which the herbal formulation may help combat infectious bronchitis in chickens, the lack of direct experimental evidence necessitates cautious interpretation. Because of the difference of the body, through clinical experiments, we can obtain more accurate data to verify the therapeutic effect of Shuanghuanglian on infectious bronchitis in chickens, and provide a more powerful basis for drug research and development. The dose of Shuanghuanglian can be controlled by the results of *in vivo* experiments to improve the shortcomings of previous theoretical research. Future research should incorporate larger clinical trials. This will help confirm the findings and enhance their relevance in practical applications (66). For example, in the future, we will deeply explore the effective components of Shuanghuanglian and the clinical application effects of these four small molecule compounds, and the types of preparations can be optimized, including soluble powder, oral liquid, injection, granules, etc. In the process of development, it is necessary to select the appropriate dosage form according to the types of animals and the characteristics of diseases, and develop a more practical veterinary treatment method through clinical trials and applied research and then verify the effects of these effective components and small molecule compounds through clinical trials.

## 5 Conclusion

This research investigates the potential of Shuanghuanglian, a traditional Chinese medicine, in treating infectious bronchitis in chickens by identifying crucial targets and pathways involved in the disease. We used a variety of analytical methods, and finally identified the potential active ingredients of Shuanghuanglian as kaempferol, quercetin, wogonin, and luteolin. The active ingredients target BCL2 and IL6 and play a therapeutic role in avian infectious bronchitis by inhibiting apoptosis and reducing inflammatory response. These findings contribute to a deeper understanding of the intricate interactions between herbal compounds and disease pathways. Furthermore, they provide a foundation for future drug development and clinical applications. Further experiments are encouraged to demonstrate the role of kaempferol, quercetin, wogonin, and luteolin.

## Data Availability

The original contributions presented in the study are included in the article/Supplementary material, further inquiries can be directed to the corresponding author.

## Footnotes

^1^ https://www.bioinformatics.com.cn/static/others/jvenn/example.html

^2^ https://www.bioinformatics.com.cn/
